# Predictors of dementia amongst newly diagnosed non-valvular atrial fibrillation patients

**DOI:** 10.1016/j.ihj.2022.11.009

**Published:** 2022-11-30

**Authors:** Akash Batta, Yash Paul Sharma, Juniali Hatwal, Prashant Panda, Budumuri Gautam Vinay Kumar, Sukhdeep Bhogal

**Affiliations:** a Department of Cardiology, Advanced Cardiac Centre, Post Graduate Institute of Medical Education & Research, Chandigarh, 160012, India; b Department of Internal Medicine, Advanced Cardiac Centre, Post Graduate Institute of Medical Education & Research, Chandigarh, 160012, India; c Department of Interventional Cardiology, MedStar Washington Hospital Centre, 110 Irving St. Suite 4B-1, Washington, NWDC, 20010, USA; d Dayanand Medical College and Hospital, Ludhiana, Punjab, 141001, India

**Keywords:** Atrial fibrillation, Dementia, Cognitive decline

## Abstract

Atrial fibrillation (AF) confers a 2-to-3-fold increased risk of developing cognitive dysfunction and dementia, independent of age and past stroke. The purpose of study was to identify risk factors for developing dementia amongst AF patients in India. This was a single-centre, prospective, observational study wherein recently diagnosed, treatment naïve, persistent non-valvular AF patients were enrolled. All patients were screened for dementia using the Mini-Mental state exam. Amongst a total of 108 patients enrolled, 40 (37%) had dementia. The most common cognitive deficits were in attention and calculation followed by memory deficits. Factors independently contributing to dementia were advanced age, female sex, presence of diabetes, elevated pulmonary artery pressures and a lower serum albumin.

## 1 Introduction

Atrial fibrillation (AF) is the most common sustained arrythmia with increasing prevalence worldwide. AF is commonly associated with various cardiac comorbidities and the incidence increases with advancing age.^1^ The prevalence of both dementia and AF increases with advancing age and hence unsurprisingly then often coexist. However, recent research has indicated that there is a high prevalence of dementia in atrial fibrillation patients independent of age and past cerebrovascular accident (CVA).^2^^,^^3^ Even after adjusting all possible confounding variables presence of AF confers a 2-to-3-fold increased risk of developing cognitive dysfunction and dementia.^4^^,^^5^

Proposed mechanisms for the same include cerebral hypoperfusion resulting from beat-to-beat cardiac output variability, reduced overall cardiac output, inflammation and endothelial dysfunction, hypercoagulability and circulatory stasis resulting in micro and macro emboli and bleeds.^6^ The end result includes grey and white matter changes resulting in axonal damage and reduced brain volume which proceeds cognitive decline.

We aimed to conduct this prospective study in anticoagulation naïve, newly diagnosed non-valvular AF patients within prior 3 months, without past transient ischemic attack (TIA) or CVA to determine the prevalence of dementia and to identify its predictors at the onset not taking into account the role of therapeutic anticoagulation.

## 2 Methods

This was a single-centre, prospective, observational study carried out in a tertiary care centre in northern India from January 2019 to December 2020. Patients diagnosed with non-valvular AF in the prior 3 months and not having received anticoagulation were enrolled for the study. All patients fulfilling the eligibility criteria were recruited and screened for dementia using the Mini-Mental state exam (MMSE).

Inclusion criteria.

1) Recently diagnosed non-valvular AF
2) Anticoagulation naïve patients
3) Persistent AF
4) Willing for participation in the study
Exclusion criteria
1) Valvular AF
2) Already on oral anticoagulation
3) Past CVA
4) Overt mood disorder or depression
5) Acute/paroxysmal AF
6) Not willing for participation in the study

### 2.1 Mini-Mental state exam

MMSE is a 30-point questionnaire that is widely used in clinical practice to determine cognitive function and screen for dementia. It is a relatively simple tool that can be applied on outpatient basis and on an average takes less than 10 min to be performed without needing specialized equipment or training. The major components of the test include examination of various neurological skills mainly-attention and calculation, registration, recall, language and orientation. The reliability and validity are excellent for diagnosing various forms of dementia and it can also be used at follow-up for assessing the change in neurological function over time.^7^ MMSE is dependent on the education level and accordingly there are variable cut-offs according to education status. Those who had dementia on initial assessment, underwent repeat MMSE at follow-up anytime between 2 and 4 weeks of the initial visit. Those who had a score lower than the education specific cut-off were diagnosed to be having dementia.

## 3 Results

A total of 108 patients were recruited. Baseline characteristics are shown in Table 1. A total of 40 (37%) patients were diagnosed to be having dementia after failing to meet the education specific MMSE cut-offs. Amongst them, 37 (34.2%) had mild cognitive dysfunction and only 3 (2.8%) had moderate cognitive dysfunction. MMSE scores of the 40 patients who had dementia were further analysed to look into the pattern of cognitive dysfunction. Most common deficits were seen in attention and calculation in 22 (55%) patients followed by deficits in working memory in 13 (32.5%). Another 7 (17.5%) patients had deficits in visuospatial skills and were unable to perform complex commands. Six (15%) patients had deficits in one or more cognitive domains.

**Table 1:** Baseline patient characteristics.

Variable	Frequency
Mean Age in years (±SD)	66.2 (±11.3)
Sex, n (%) (n = 108)	
Female	39 (36.1%)
Male	69 (63.9%)
Mean BMI (kg/m^2^) (±SD)	24.8 (±3.54)
**Co-morbidities, n (%) (n = 108)**	
Diabetes mellitus	33 (30.6%)
Hypertension	75 (69.4%)
Coronary artery disease	23 (21.3%)
Dyslipidemia	26 (24.1%)
Chronic kidney disease	22 (20.4%)
Obesity (BMI ≥25 kg/m^2^)	42 (38.9%)
Smoking	20 (18.5%)
Alcohol intake (>2 drinks/day)	40 (37%)
CHA_2_DS_2_VASc score	3.0 (±1.3)
HAS-BLED score	1.8 (±1.2)
**Echocardiographic parameters n (%) (n = 108)**	
LVEF classification	
Normal LVEF (50–70%)	44 (40.7%)
Mild LV systolic dysfunction (40–50%)	13 (12%)
Moderate LV systolic dysfunction (30–40%)	16 (14.8%)
Severe LV systolic dysfunction (<30%)	35 (32.4%)
Mean LVEF (%) (±SD)	45.8 (±14.1%).
Mean PASP (mm Hg) (±SD)	38.7 ± 9.8%
**Laboratory parameters, n (%) (n = 108)**	
Anemia	50 (46.3%)
Hemoglobin (gm/dL ± SD)	12.6 (±1.8)
Mean creatinine (mg/dL ± SD)	1.3 (±1.1)
Urea (mg/dL ± SD)	42.6 (±26.5)
Mean albumin gm/dL ± SD)	3.9 (±0.4)
**Anticoagulation data, n (%) (n = 108)**	
CHA_2_DS_2_-VASc ≤ 1	14 (13%)
CHA_2_DS_2_-VASc ≥2	94 (87%)
Appropriate anticoagulation use (CHA_2_DS_2_-VASc ≥2)	74/94 (78.7%)
Total patients receiving oral anticoagulation, **n (%) (n = 74)**	
Total NOACs prescription	48 (64.9%)
- Dabigatran	34 (70.8%)
- Rivaroxaban	8 (10.8%)
- Apixaban	6 (8.1%)
Vitamin K antagonists	26 (35.1%)

All values are presented as the n (%). Continuous variables were presented as mean ± S.D. **Abbreviation:** BMI, body mass index; LVEF, left ventricular ejection fraction; NOACs, newer oral anticoagulants.

Clinical, laboratory and echocardiographic parameters were analysed for association with dementia as shown in Table 2. On univariate analysis, factors significantly linked to dementia at presentation were advanced age, female sex, higher BMI, presence of diabetes, underlying chronic kidney disease, a higher CHA_2_DS_2_VASc score, a higher HAS-BLED score, elevated pulmonary artery systolic pressure, a lower serum albumin and anemia.

**Table 2:** Demographic, echocardiographic and laboratory parameters affecting dementia.

Variable	Dementia (n = 40)	No dementia (n = 68)	P- value
Age, years, mean (±SD)	71.6 (±7.9)	62.9 (±11.8)	**0.001**
BMI, (kg/m^2^) mean ± (SD)	26.2 ± 3.6	24 ± 3.3	**0.001**
Sex, n (%)			
Male	17 (41.5%)	52 (77.6%)	**0.001**
Female	24 (58.5%)	15 (22.4%)	
Diabetes, n (%)	20 (48.8%)	513 (19.4%)	**0.002**
Hypertension, n (%)	31 (75.6%)	44 (65.7%)	0.38
CAD, n (%)	9 (22%)	14 (20.9%)	0.89
Dyslipidaemia, n (%)	14 (34.1%)	12 (17.9%)	0.06
Smoking, n (%)	7 (17.1%)	13 (19.4%)	0.76
Alcohol intake, n (%)	11 (26.8%)	29 (43.3%)	0.08
CHA_2_DS_2_-VASc score, mean ± (SD)	3.5 ± 1.1	2.7 ± 1.3	**0.001**
HAS-BLED score, mean ± (SD)	2.3 ± 0.8	1.6 ± 1.3	**0.001**
Echocardiographic parameters			
LVEF, %, mean ± (SD)	44.3 ± 15.6	46.8 ± 13.2	0.43
PASP, mm Hg; mean ± (SD)	43.1 ± 9.7	36 ± 9	**<0.001**
Mitral regurgitation, n (%)			
None	17 (41.5%)	40 (59.7%)	0.12
Mild	15 (36.6%)	15 (22.4%)	
Moderate	4 (9.8%)	9 (13.4%)	
Severe	5 (12.2%)	3 (4.5%)	
Laboratory parameters			
Mean Hemoglobin (gm/dL ± SD)	12.1 ± 2	12.9 ± 1.7	**0.01**
Mean Platelet count (lacs/dL SD)	2.22 ± 0.98	2.03 ± 0.8	0.60
Mean creatinine mg/dL ± SD)	1.5 ± 1.4	1.2 ± 0.8	0.42
Mean urea mg/dL ± SD)	42.3 ± 22.6	42.8 ± 28.8	0.31
Mean albumin gm/dL ± SD)	3.8 ± 0.4	4 ± 0.4	**0.01**

**Abbreviation:** BMI; body mass index, LVEF; left-ventricular ejection-fraction, CAD; coronary artery disease, PASP; pulmonary artery systolic pressure.

**Fig. 1:**
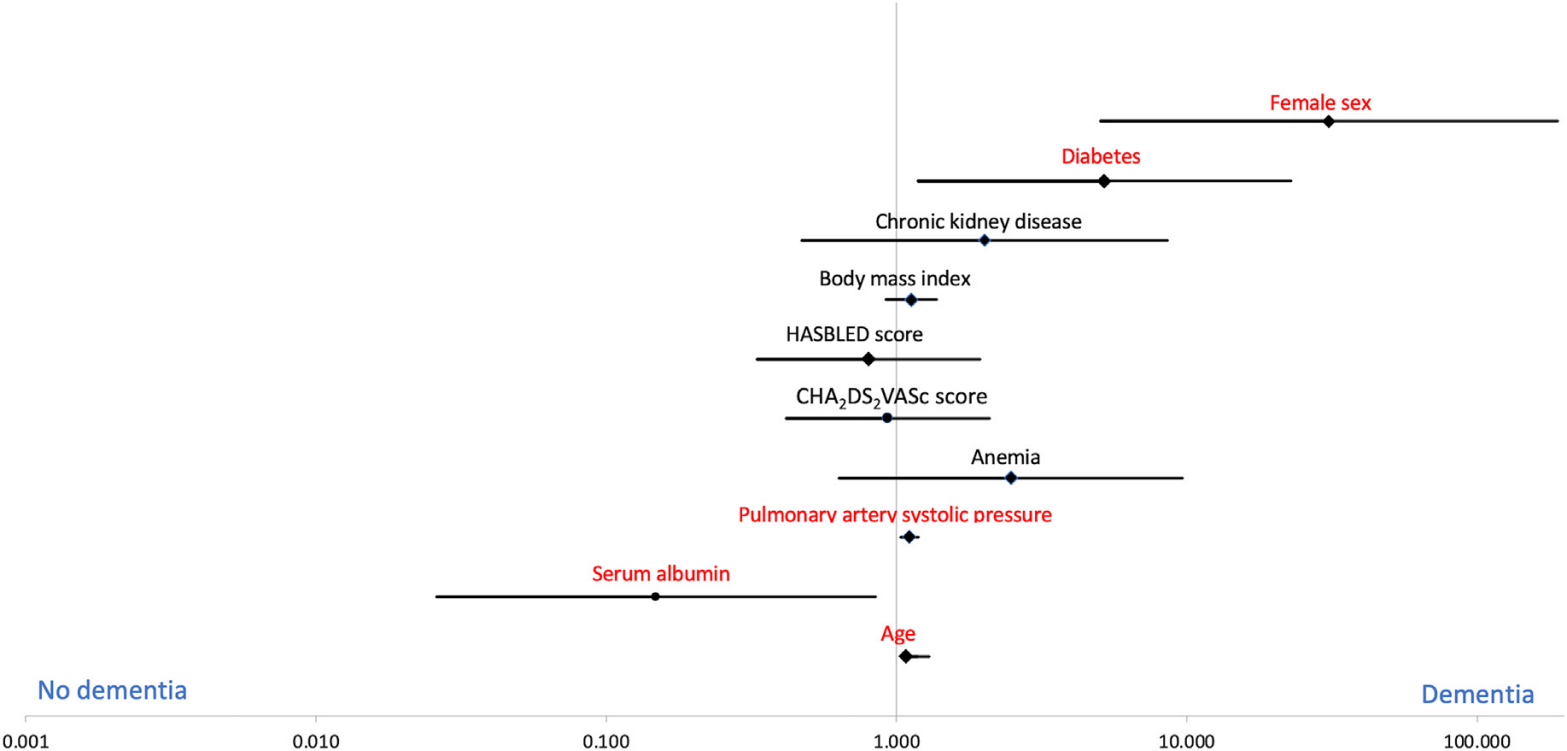
Odds ratio plot of logistic regression demonstrating the 95% confidence intervals for dementia in atrial fibrillation patients. Factors independently contributing to dementia at presentation included advanced age, female sex, presence of diabetes, elevated pulmonary artery systolic pressure at presentation and a lower serum albumin.

All these parameters had p < 0.05 and were further evaluated using binary logistic regression to determine the parameters which had an independent association with dementia (Table 3). Factors independently linked to dementia on regression analysis included advanced age (OR: 1.182, 95% CI: 1.075–1.299; p = 0.001), female sex (OR: 30.895, 95% CI: 5.039–189.422; p = 0.001), presence of diabetes (OR: 5.188, 95% CI: 1.180–22.811; p = 0.03), elevated pulmonary artery systolic pressure at presentation (OR: 1.109, 95% CI: 1.034–1.299; p = 0.0304) and a lower serum albumin (OR: 0.148, 95% CI: 0.026–0.849; p = 0.03). The relationship of each variable with dementia has been shown on the odds ratio plot of logistic regression in Fig. 1.

**Table 3:** Binary logistic regression analysis.

Risk factors	Multivariable analysis		
	OR	CI (95%)	P-value
Age	1.182	1.075–1.299	**0.001**
Female sex	30.895	5.039–189.422	**0.001**
Diabetes	5.188	1.180–22.811	**0.03**
Chronic kidney disease	2.009	0.470–8.592	0.35
BMI	1.121	0.917–1.374	0.26
CHA_2_DS_2_-VASc score	0.934	0.417–2.093	0.87
HAS-BLED score	0.801	0.331–1.938	0.62
Anemia	2.478	0.635–9.665	0.19
PASP	1.109	1.034–1.299	**0.004**
Serum Albumin	0.148	0.026–0.849	**0.03**

**Abbreviation:** BMI; body mass index, PASP; pulmonary artery systolic pressure.

## 4 Discussion

Dementia not only leads to worse quality of life, but also confers excess morbidity and mortality.^8^ Factors that have been associated with increased dementia in AF include diabetes, increased duration of AF, higher CHA_2_DS_2_VASc score, decreased time in therapeutic anticoagulation and past CVA.^6^^,^^9^ Most of the risk these results were derived from retrospective observational studies and registry data. In our study, we looked into various risk factors for dementia at the time of diagnoses which has been seldom studied before.

Estimated prevalence of dementia in American population is around 14% at age 70 or more. Data from Indian subcontinent is scant, but limited research suggests it is less than the western counterparts at around 8% at 75 years. Another important finding is the fact that dementia occurs at a younger age among Indians compared to western population despite the lower prevalence.^10^ Studies on dementia prevalence in Indian AF patients are not available and our study is the first in this regard which clearly demonstrates the excess prevalence of dementia in Indian AF patients compared to general population.

The association with advanced age and dementia is understandable as aging involves neuronal apoptosis and reduced cerebral volume. Another important finding in our study was the predisposition of the female gender to develop dementia compared to the male population. Our findings are in line with a recent large study which confirmed that the risk of dementia was higher in females and the differences were largely believed to stem from sex hormones and their receptor signalling (oestrogen and androgen). Female sex has a higher propensity to develop Alzheimer's dementia also which also is a result of sex-related deposition of arrhythmogenic substrate in the atrial tissue leading to increased incidence of AF and subsequent dementia.^11^ Diabetes has been linked to dementia in the absence of AF.^12^ The two when present have shown to accelerate the atherogenicity and thromboembolic potential throughout the cardiovascular system. Another important association is the associated microvascular dysfunction in diabetes which is compounded by AF which further leads to cerebral hypoperfusion and gradual cognitive decline.^13^ Elevated pulmonary pressures have shown to contribute to cognitive decline and dementia amongst pulmonary arterial hypertension (PAH) patients, however the relationship of pulmonary pressures with dementia in AF patients is less studied.^14^ Theories contributing to cognitive decline amongst PAH patients include increased prevalence of concomitant anxiety and depression, sub-clinical hypoxemia and worse quality of life due to reduced functional status and increased cardiac symptoms in this population.^14^ The last parameter which in our study that had independent relationship with dementia was a lower serum albumin levels. Albumin is a simple and widely accepted marker of a person's nutritional status and contributes to excess morbidity and mortality across the entire spectrum of cardiovascular disease.^15^^,^^16^ Low albumin have previously linked to increased incidence of Alzheimer's and all cause dementia.^17^ A lower serum albumin reflects poor overall health of an individual. Whether serum albumin is a bystander along with other nutritional deficiencies in frail elderly patients which may contribute to dementia or it has an independent role in pathogenesis is yet to be determined.

A key step in prevention of dementia amongst AF patients is the timely institution of effective oral anticoagulation. The same has been realized in large recent prospective studies.^18^ However, prescription rates and adherence to anticoagulation is an unmet need with dismal figures showing a large gap between knowledge and actual practice. Overall only around half of AF patients who merit anticoagulation receive therapeutic anticoagulation with acceptable time in therapeutic range. Scarce data from India also supports this observation and the rates of therapeutic anticoagulation is probably lower.^19^^,^^20^ NOACs have improved the patient adherence rates because of little drug interactions, lesser rates of bleeding and no requirement of period coagulation parameter testing compared to VKAs.^21^ The impact of oral anticoagulation on dementia could not be evaluated in our study due to the nature of the study and this was a limitation of our study. Still effective anticoagulation remains the most favoured approach to lessen the burden of dementia amongst AF patients as of today.

While there are certain merits of our study, we must also accept the limitations of our study. The major limitations of our study included lesser patient number for a common disease (only newly diagnosed anticoagulation naïve AF patients) due to stringent selection criteria, lack of follow-up and absence of neurological imaging to correlate the minor and major vascular events with cognitive dysfunction. A comparative arm was lacking which would have further strengthened the findings of this study.

## 5 Conclusion

The prevalence of dementia is increased amongst AF patients independent of prior CVA. Dementia is widely prevalent among newly diagnosed AF patients in India with 37% of all patients having dementia at presentation. Factors independently contributing to dementia in our study included advanced age, female sex, presence of diabetes, elevated pulmonary artery systolic pressure at presentation and a lower serum albumin.

## Declarations

### Conflicts of interest

None.

No financial relationships.

### Patient consent

Written informed consent obtained from all participants.

### Ethical approval

The study protocol conforms to the ethical guidelines of the declaration of Helsinki and was reviewed and cleared by the ethical committee of the institute.
